# Primary multiple osseous leiomyosarcomas of the spine metastasizing to the skull

**DOI:** 10.11604/pamj.2016.24.334.8297

**Published:** 2016-08-30

**Authors:** Oudrhiri Mohammed Yassaad, Raouzi Nabil, El KacemiInas, Allaoui Mohammed, Arkha Yasser, El Ouahabi Abdessamad

**Affiliations:** 1 Neurosurgery Department, Hôpital des Spécialités, UHC Ibn Sina, Rabat, Morocco; 2 Neuropathology Department, Hôpital des Spécialités, UHC Ibn Sina, Rabat, Morocco

**Keywords:** Leiomyosarcoma, spine, primary, spinal cord compression, immunohistochemistry

## Abstract

Primary osseous leiomyosarcoma of the spine is a very unusual condition, with only few cases being reported in the literature. In fact, this type of tumors arises from the smooth muscle cells and occurs usually in the uterus and the gastrointestinal tracts. If the spine should be involved, it occurs generally as a metastatic location. Location to the spine as a primary site is exceedingly rare. We present the case of a 37 years old female patient, with multiple spine levels involvement - to vertebral body and to posterior aspects of Vertebra, causing spinal cord compression syndrome. A secondary location to the skull was diagnosed one month later. Through a literature review, we analyze various aspects in the diagnosis and management of this rare entity.

## Introduction

Primary osseous leiomyosarcoma of the spine is a very rare condition. This type of tumors arises from the smooth muscle cells and occurs usually in the uterus and the gastrointestinal tracts. The spine may then be involved as a metastatic location [1]. Primary location to bone is quite unusual, and has been mainly reported on long bones of the limbs [2]. Location to the spine as a primary site is exceedingly rare.

## Patient and observation

**Figure 1:**
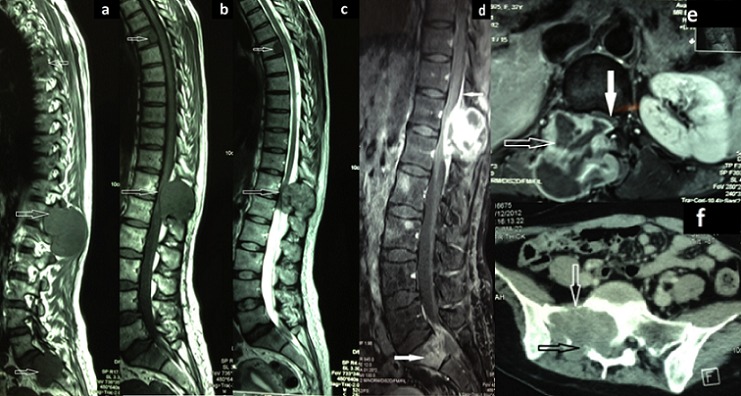
MRI images of the spine in sagittal T1 (a&b), sagital T2 (c) and post contrast sagital (d) and axial (e). The tumor is hypointense to bone on T1 weighted images (a&b), slightly hyperintense and heterogeneous on T2 weighted images (c), with intense, irregular contrast enhancement (d&e). Note the vertebral body involvement in T5 lesion (superior arrows in a, b & c). Note the severe spinal cord compression (middle arrows in a, b, c & e). Note the contrast enhancement along the dura that extends upward and downward (upper arrow in d). Image “f” is an axial CT scan slide showing the sacral lesion with the bone lysis (arrow).

**Figure 2:**
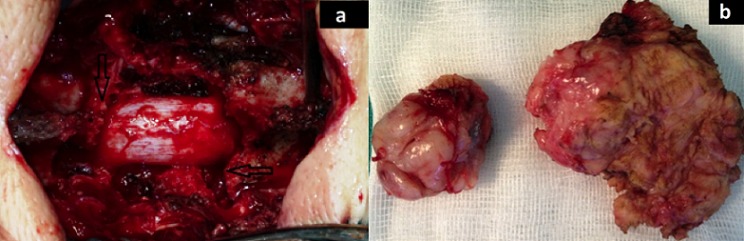
Operative findings. The tumor caused extended bone lysis (arrows in a); the dura was not invaded. The tumor was white-reddish and multilobulated (b)

A 37 years old female was admitted to our department for a 2 months history of lombosacral radiculopathy - especially to the right leg - and low back pain, which was treated with NSAID (non steroid anti-inflammatory drug) with only partial remission. Two weeks before admission, the patient experienced rapidly progressive paraparesis with urinary dysfunction. Her past medical history was unremarkable. On admission, the patient was in moderate general condition (Karnovsky 50%) with a predominant right paraparesis (3/5), a sensory level below the umbilicus and genitor-urinary sphincter dysfunction. Reflexes were exaggerated on lower limbs. Local examination of the spine could find tenderness in the right paravertebral area on T12-L1 level, related to a subcutaneous fixed mass. Computed tomography and magnetic resonance imaging of the spine were performed. Findings were compatible with multiple metastases, and involved: (Figure 1) the posterior part of the vertebral body of T5, with posterior wall rupture (Figure 1 b); the posterior elements of T12 and L1 vertebras, with a tissular mass causing bone lysis, severe cord compression and extending to the paravertebral region; the vertebral body was intact (Figure 1 a, b, c, d, and e). Vertebral body of S1, with lysis, right spinal canal invasion and extension to the pre-vertebral area (Figure 1 f) These lesions were iso- to hypo-intense to bone on T1 weighted images (Figure 1 a, b), and heterogeneous on T2 weighted images with multiple hyperintense foci (Figure 1 c). Contrast enhancement was intense and also heterogeneous, and was extending vertically along the epidural space of the medial lesion (Figure 1 d). On CT scan, bone lysis was observed. Computed tomography of brain, cervical, thoracic, abdominal and pelvian regions, and also systemic investigations were all negative for a primary tumoral lesion. The patient was proposed for a posterior decompression on T12-L1 level with instrumented fixation -as symptoms were mainly related to it- and also a sacral decompression. In the dorsolumbar area, the tumor was immediately found under the subcutaneous tissue, invading the paravertebral muscles: A multi-lobulated firm mass, with a white-reddish appearance, mainly avascular. The tumor was not encapsulated but could easily be differentiated and separated from the surrounding tissue. The bone lysis was evident, and the spinal cord compression severe. The dura matter was not infiltrated (Figure 2). Dural decompression was completed and T11-L2 screw fixation was performed - given the pedicle lysis on CT-scans - after gross total removal was achieved.

**Figure 3:**
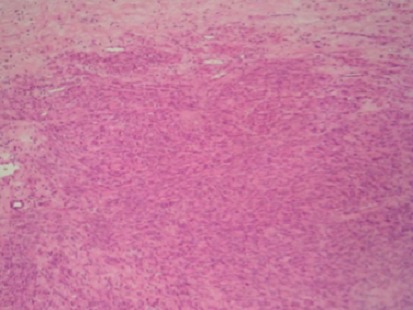
Photomicrograph of the biopsy specimen showing interlacing fascicles of spindle-shaped cells with elongated or blunt-ended nuclei and eosinophilic cytoplasm. Hyperchromatic or pleomorphic nuclei and mitoses are occasionally observed. (Haematoxylin and eosin; Original magnification, x100)

**Figure 4:**
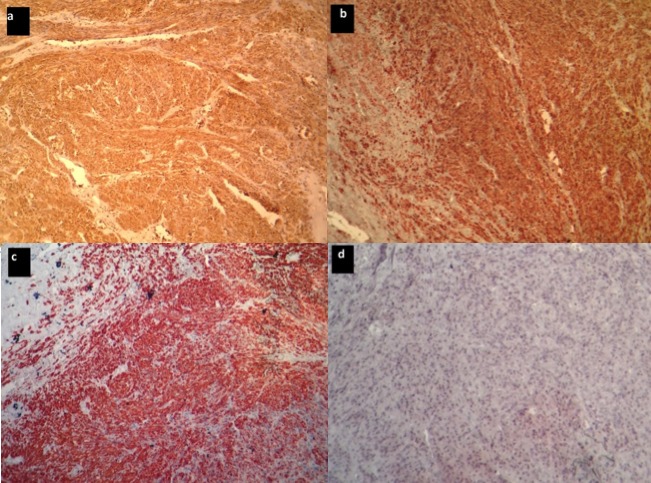
Immunohistochemistry staining: the tumor cells are positive for H-Caldesmon (a), Alpha-Smooth Muscle Actin (b), Desmin (c), negative for S100 (d), (Original magnification, x100)

**Figure 5:**
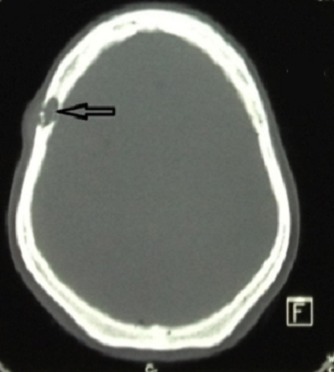
Axial CT scan slide, showing the metastatic frontal bone lytic lesion (arrow)

Taking advantage of the patient’s position, and given the history of lombosacral radiculopathy, an S1 right hemilaminectomy with curettage of the sacral body and radicular decompression were also performed. Operative findings were compatible with the upper level. Histology specimens were analyzed for both localizations, with an identical result: a malignant spindle cell proliferation with large necrotic areas. Tumor cells were arranged into short fascicles and interlacing bundles (Figure 3). The nuclei were hyperchromatic and occasionally highly pleiomorphic. Mitotic figures were recognized in about 15 per 10 high power fields. Large necrotic areas were observed (about 50% of the tumor surface). Immunohistochemical staining was positive for smooth muscle actin, desmin and H-caldesmon, and negative for S100 protein, and CD34 (Figure 4). Focal reactivity was observed with EMA. Thus, the diagnosis of a leiomyosarcoma grade 3 of the FNCLCC was established. In the postoperative period, the patient started improving her motor score, and gaining control on her urinary function. She was discharged on both rehabilitation unit and oncology unit for adjuvant therapy. One month after discharge, the patient presented for persistent headaches, with no focal signs. Locally, we could find tenderness on the right frontal area of the skull convexity. CT scan was performed, showing a small lytic frontal bone lesion extending to the subcutaneous tissue without dural extension (Figure 5). A small craniectomy around was performed, and the histopathology came back with no surprise. At one year follow up, the adjuvant therapy failed to obtain tumor control. The thoracic and abdominopelvian CT scan showed both local and systemic progression. Although her motor function improved continuously to become self dependent, her general condition was worsening. She was then assessed for palliative treatment. The patient died two years after her initial management.

## Discussion

Leiomyosarcoma is a malignant neoplasm originating from the smooth muscle cells. It occurs predominantly in the retroperitoneum, the intrabdominal space and the subcutaneous tissue of the extremities [3]. Primary location to bone was mainly described for long bones such as femur and tibia [2], and account for 0.1% to 0.64% of all primary bone tumors [3]. The osseous leiomyosarcoma is thought to originate from the smooth cells in the vasculature. Other hypothesis includes a multipotential mesenchymal cell or an intermediate cell capable of smooth muscle cell differentiation [2]. This osseous origin can only be assessed after careful body screening to rule out a usual primary location, as osseous metastases may be the initial manifestation of primary uterine leiomyosarcoma [4].

### Literature review

We performed a careful analysis of the existing literature through a Pubmed research of Medline’s database (using the keywords: primary, leiomyosarcoma, spine, and skull). To date, 17 cases of primary spine locations have been reported (Table 1). These locations included both the vertebral body [1–3, 5–12] and the posterior aspects of the vertebra [13–15]. Potsi, in his recent paper [1], took only into account the vertebral body involvement and found 14 reported observations (including his work). We think that the development into the posterior elements of the vertebra and the paravertebral area may be considered as well. In fact, our patient presented both aspects: a vertebral body lesion and a posterior vertebral/paravertebral lesion. The skull manifestation described here was considered as metastatic from the spine. No publication described primary multiple spine level involvement as was the case with our patient. Central nervous system involvement was described mainly as intracranial tumors [10, 16]. This tumor affects adult patients, with ages ranging from 23 years to 75 years. No case has yet been described into the pediatric population. Unlike other bone locations where there is no sex preponderance[8], primary spinal locations occur more frequently in women (13 out of 18) as for leiomyosarcoma [11]. Most patients presented with pain and signs of spinal cord compression and progressive myelopathy. Leiomyosarcoma of the spine presents as an ill-defined lytic mass, affecting both the vertebral body and/or the posterior aspects, invading the spinal canal. A prevertebral or a paravertebral development is usually seen. A compression fracture may also be seen [11]. The spine is uniformly affected. Although there is no specific radiologic criterion, this tumor appears frequently iso- to hyperintense to bone on T1 weighted images; a heterogeneous hyperintensity is seen on T2 weighted images with irregular enhancement [1]. Histopathology should differentiate leiomysarcoma from other spindle cell tumors. In this concern, immunohistochemical study is very useful: a positive reactivity with smooth muscle actin, vimentin and caldesmon, but negative reactivity for S100 protein and CD34 [3, 11]. Reactivity for Desmin is irregular [1, 9, 12, 15]. Some special histopathological aspects as an epithelioid differentiation were described [15].

**Table 1:** Summary of published cases of primary spinal leiomyosarcomas

Case / Year	Age / Sexe	Location	Particularity	Treatment	Outcome / Follow up
Berlin et al. 1987 [5]	70 / F	Sacroiliac	----	RT	DOD at 20 months
Lo et al. 1995 [8]	39 / M	T8	Hemorrhagic	Surgery + RT	----
Antonescu et al. 1997 [2]	36 / M 42 / F 64 / F 64 / F	L2 Sacrum Sacroiliac Sacroiliac	Radiation Induced ---- ---- ----	Surgery + RT Biopsy Resection Resection	DOD at 6 months ---- Recurrence at 5 months DOD at 12 months
Ochiai et al. 2000 [9]	69 / M	C7	----	Biopsy + RT	DOD at 6 months
Ritter et al. 2000 [10]	35 / F	T3-T4	AIDS related	Surgery + RT	----
Nishida et al. 2002 [3]	47 / F	L2	----	Surgery + RT	FOD at 25 months
Krepler et al. 2002 [7]	45 / M	T7	----	Surgery	DOD at 24 months
Ganau et al. 2002 [6]	23 / F	Sacroiliac	----	Biopsy + Curetage + RT	----
Aksoy et al. 2002 [13]	70 / F	T2 (vertebral body and post. elements)	----	Surgery	----
Sasaguri et al. 2004 [11]	75 / F	T12	Compression fracture	Surgery	FOD at 4 months
Marshman et al. 2005 [15]	61 / F	C3-C5 (post. elements and paravertebral)	----	Surgery	----
Lehman et al. 2007 [14]	45 / M	C1-C2 (post. elements and paravertebral)	----	Surgery	----
Potsi et al. 2009 [1]	57 / F	T11	----	Surgery	FOD at 6 months
Sucu et al. 2011 [12]	25 / F	C2	----	Surgery	FOD at 12 months
Present case	37 /F	T12-L1 post. elements and paravetebral T5 vertebral body S1 vertebral body	Multiple	Surgery	DOD at 24 months

Abreviations: F: Female; M: Male; RT: Radiotherapy; DOD: Dead of disease; FOD: Free of disease; AWD: Alive with disea

Although no consensus about treatment is available, management of these tumors, given their malignancy, should include total radical excision whenever possible [1]. Adjuvant therapy is mandatory and may include radiotherapy alone or an association to chemotherapy. In our case, we first considered the metastatic origin of the tumors given the multiple locations. When a primary lesion has been ruled out after careful repeated systemic screenings, the treatment modality consisted on a surgical treatment of the symptomatic level through a posterior approach, radical excision of the tumor and posterior fixation. Surgical excision of all lesions was not considered given the initial spreading of the tumor.

## Conclusion

The diagnosis of a primary spine location of leiomyosarcoma is an unusual condition. It should only be made after careful screening for a primitive tumor, and on immunohistochemical basis. Surgical treatment, as for other spine malignancies, should be as radical as possible. We report herein another unusual aspect of this tumor.
